# Cold Atmospheric Plasma, Created at the Tip of an Elongated Flexible Capillary Using Low Electric Current, Can Slow the Progression of Melanoma

**DOI:** 10.1371/journal.pone.0169457

**Published:** 2017-01-19

**Authors:** Y. Binenbaum, G. Ben-David, Z. Gil, Ya. Z. Slutsker, M. A. Ryzhkov, J. Felsteiner, Ya. E. Krasik, J. T. Cohen

**Affiliations:** 1 Laboratory of Applied Cancer Research, Rambam Healthcare Campus, Haifa, Israel; 2 Rappaport Faculty of Medicine, Technion-Israel Institute of Technology, Haifa, Israel; 3 Laboratory of Plasma Physics, Physics Department, Technion-Israel Institute of Technology, Haifa, Israel; Universite Toulouse III Paul Sabatier, FRANCE

## Abstract

**Introduction:**

Cold Atmospheric Plasma Jet (CAPJ), with ion temperature close to room temperature, has tremendous potential in biomedical engineering, and can potentially offer a therapeutic option that allows cancer cell elimination without damaging healthy tissue. We developed a hand-held flexible device for the delivery of CAPJ to the treatment site, with a modified high-frequency pulse generator operating at a RMS voltage of <1.2 kV and gas flow in the range 0.3–3 l/min. The aims of our study were to characterize the CAPJ emitted from the device, and to evaluate its efficacy in elimination of cancer cells in-vitro and in-vivo.

**Methods and Results:**

The power delivered by CAPJ was measured on a floating or grounded copper target. The power did not drastically change over distances of 0–14 mm, and was not dependent on the targets resistance. Temperature of CAPJ-treated target was 23°-36° C, and was dependent on the voltage applied. Spectroscopy indicated that excited OH^-^ radicals were abundant both on dry and wet targets, placed at different distances from the plasma gun. An in-vitro cell proliferation assay demonstrated that CAPJ treatment of 60 seconds resulted in significant reduction in proliferation of all cancer cell lines tested, and that CAPJ activated medium was toxic to cancer cells. In-vivo, we treated cutaneous melanoma tumors in nude mice. Tumor volume was significantly decreased in CAPJ-treated tumors relatively to controls, and high dose per fraction was more effective than low dose per fraction treatment. Importantly, pathologic examination revealed that normal skin was not harmed by CAPJ treatment.

**Conclusion:**

This preliminary study demonstrates the efficacy of flexible CAPJ delivery system against melanoma progression both in-vitro and in-vivo. It is envisioned that adaptation of CAPJ technology for different kinds of neoplasms use may provide a new modality for the treatment of solid tumors.

## Introduction

Cold atmospheric plasma jet (CAPJ) has become extremely attractive for biological applications, due to its potential in disinfection, wound healing and cancer treatment [1]. This type of plasma can be generated by different types of gas discharges, for instance by either inductively or capacitively coupled RF discharges, corona discharge and dielectric barrier discharge (DBD). DBD discharge is used in most plasma guns producing CAPJ. Namely, the plasma formation occurs due to capacitive coupling between the high-voltage electrode and the plasma generated by ionization of the gas flow at atmospheric pressure. This plasma can be considered as a low-ionized, collisional non-thermal plasma with temperature of ions and neutrals of ~300 K. Several recent review papers [2,3] describe different geometries of the plasma guns and power supplies used for CAPJ generation, and the parameters of the plasma measured by different electrical, optical and spectroscopic diagnostics.

CAPJ can be generated using pulse power supplies operating at frequencies in the range 1–30 kHz in noble gas or noble gas mixture (as Ar + air [4], Ar + N_2_ [5,6], He [3,7,8]). The application of these gases is related, following Paschen dependencies, to decreased breakdown threshold allowing application of voltage pulses with amplitude ≤5 kV [9–13]. For instance, Wang *et al*. [7] used HV pulses with amplitude of ~3.7 kV and variable duty cycle for generating DBD discharges in He gas with flow rate of 4.6 l/min. Graves[14] operated with DBD discharges in noble gases with ≤1% admixture of N_2_ and/or O_2_ at flow rate of 3 l/min at frequencies in the range 1–30 kHz and voltage amplitude down to ~1.9 kV, and Shashurin *et al*. [3] applied HV pulses at repetition rate of 25 kHz with even lower voltage amplitude of 1.5 kV but with gas flow rate increased up to 11.5 l/min to obtain DBD discharge in He gas.

Since 2004, when it was shown that CAPJ causes mammalian cells to detach from culture surfaces [15], culminating evidence has indicated that CAPJ has significant effect on cell biology. It is now clear that CAPJ affects cells in a dose-dependent manner. Namely, very low doses affect cellular motility, integrin expression and membrane lipid peroxidation; higher doses induce stress-related apoptosis; and very high doses cause necrosis [16]. It is also evident that CAPJ mediates its action by reactive oxygen species (ROS), which lead to downstream DNA damage [17]. The ability of CAPJ to induce DNA damage and apoptosis has attracted oncological research of CAPJ treatment on numerous cancerous cell types, such as pancreatic cancer [18], melanoma [19], glioblastoma [20], neuroblastoma [21], lung cancer [22], breast cancer [23], colon cancer [24] and hepatocellular carcinoma [23]. While very few of the experiments were done in-vivo, CAPJ is gaining ground as a promising new oncological modality since it appears to be more potent against tumor cells rather than non-neoplastic cells [25]. Medical application of CAPJ, however, should take into account potential risks of the technology to the human body. These may include high temperature, high voltage, poisonous gases, ultraviolet (UV), soft x-rays and other electromagnetic radiation which can accompany CAPJ generation, and should be either avoided or minimized [26]. Since 2004, when it was shown that CAP causes mammalian cells to detach from culture surfaces [15], culminating evidence has indicated that CAP has significant effect on cell biology. It is now clear that CAP affects cells in a dose-dependent manner. Namely, very low doses affect cellular motility, integrin expression and membrane lipid peroxidation; higher doses induce stress-related apoptosis; and very high doses cause necrosis [16]. It is also evident that CAP mediates its action by reactive oxygen species (ROS), which lead to downstream DNA damage [17,27]. The ability of CAP to induce DNA damage and apoptosis has attracted oncological research of CAP treatment on numerous cancerous cell types, such as pancreatic cancer [18], melanoma [19], glioblastoma [20], neuroblastoma [21], lung cancer [22], breast cancer [23], colon cancer [24] and hepatocellular carcinoma [23]. While very few of the experiments were done in-vivo, CAP is gaining ground as a promising new oncological modality since it appears to be more potent against tumor cells rather than non-neoplastic cells [25]. Medical application of CAP, however, should take into account potential risks of the technology to the human body. These may include high temperature, high voltage, poisonous gases, ultraviolet (UV), soft x-rays and other electromagnetic radiation which can accompany CAP generation, and should be either avoided or minimized [26].

Melanoma is the most aggressive skin cancer and the sixth most common cancer in North America. Most cases of malignant melanoma are diagnosed at an early stage, when surgical excision can be curative. In some cases, however, the tumor cannot be completely excised due to its proximity to vital anatomical structures such as nerves and blood vessels [28]. In these cases, where drug treatment or radiation therapy are mandated, CAPJ treatment may be an attractive option. Several studies describing CAPJ effect on melanoma cells have been published so far, but none of them tested the technology in-vivo [19,23,29–33]. A major technical challenge that restricted the adaptation of CAPJ technology to the clinical setting is the inability to easily maneuver the plasma plume over the treatment site. Since most described CAPJ generation devices have diameter >5 mm and length of several cm, their integration in the typical surgical routine is limited, especially in situations that necessitate extreme delicacy or insertion of the device into small cavities. To overcome this obstacle, attempts have been made to transport the plume from the CAPJ generating unit to the treatment location. Recent experimental research [34] showed that CAPJ can be transported along distances ≥1 m inside dielectric channels in the form of a "bullet" which acquires a potential almost equal to the high-voltage electrode potential. The latter results in a high electric field at the head of the “bullet” allowing its propagation in dielectric channel. However, this method cannot be used when the device is in contact with the human body because of the increased stray capacitance between the plasma bullet and the body. This results in decrease of the “bullet” potential and, respectively leads to the electric field decrease, which in turn stops the bullet propagation. To overcome this problem, CAPJ should be generated at the vicinity of the treatment location, rather than being delivered distally. For surgeons to adopt the technology, the dimensions of the plasma source should be minimized to mm-scale, it should not be influenced by direct contact with the human body (patient or surgeon), and it should be flexible, maneuverable and safe. Melanoma is the most aggressive skin cancer and the sixth most common cancer in North America. Most cases of malignant melanoma are diagnosed at an early stage, when surgical excision can be curative. In some cases, however, the tumor cannot be completely excised due to its proximity to vital anatomical structures such as nerves and blood vessels [28]. In these cases, where drug treatment or radiation therapy are mandated, CAP treatment may be an attractive option. Several studies describing CAP effect on melanoma cells have been published so far, but none of them tested the technology in-vivo [19,23,29–33]. A major technical challenge that restricted the adaptation of CAP technology to the clinical setting is the inability to easily maneuver the plasma plume over the treatment site. Since most described CAP generation devices are bulky, their integration in the typical surgical routine is limited, especially in situations that necessitate extreme delicacy or insertion of the device into small cavities. To overcome this obstacle, attempts have been made to transport the plume from the CAP generating unit to the treatment location. Recent experimental research [34] showed that CAP can be transported along distances ≥1 m inside dielectric channels in the form of a "bullet" which acquires a potential almost equal to the high-voltage electrode potential. The latter results in a high electric field at the head of the “bullet” allowing its propagation in dielectric channel. However, this method cannot be used when the device is in contact with the human body because of the increased stray capacitance between the plasma bullet and the body. This results in decrease of the “bullet” potential and, respectively leads to the electric field decrease, which in turn stops the bullet propagation. To overcome this problem, CAP should be generated at the vicinity of the treatment location, rather than being delivered distally. For surgeons to adopt the technology, the dimensions of the plasma source should be minimized to mm-scale, it should not be influenced by direct contact with the human body (patient or surgeon), and it should be flexible, maneuverable and safe.

The purpose of this study was to evaluate the efficacy of our newly developed CAPJ device on melanoma in-vitro and in-vivo, and to evaluate the user-device interaction of this novel CAPJ generation device in clinical setting.

## Materials and Methods

### Experimental setup

**Fig 1:**
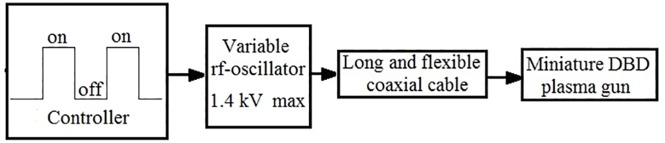
Block scheme of the RF generator and plasma gun for CAPJ generation.

In the case of DBD discharges, the plasma generation occurs mainly during the rising and falling time of the applied voltage pulse [35]. In order to improve the efficiency of the plasma generation, the repetition rate of the voltage pulses in our research was increased to RF frequency range (>1 MHz). These pulses were applied by bursts with duration in the range of 4×10^−4^–8×10^−4^ s and repetition rate 150–600 Hz adjusted by electronic scheme. This was used to change the duty cycle of the application of these bursts in the range (0.03–0.5). The plasma parameters were controlled by the change of the value of the voltage pulses, the duration of bursts and by the duty cycle of the pulse generator operation. The latter allows us to change the average power delivered to the plasma plume within the range 0.5 – 3W, depending on the treatment requirements. The RF pulse generator is connected to plasma guns having different geometries by a commercial, thin and flexible, coaxial cable with low dielectric losses (voltage breakdown of 2.5 kV) whose lengths can be varied depending on the requirement of the experiment and the research carried out. This cable also served as a resonance capacitor in the high-voltage part of the RF generator thus providing a high efficiency of the power delivering to the plasma gun. In addition, the shielding of the coaxial cable decreases electromagnetic radiation (~70 dB) and eliminates electrical shock in the case of cable electrical breakdown. The generator produces RF pulses with RMS voltage up to 1.4 kV (see block scheme of the generator in Fig 1) but in the experimental research with CAPJ treatment, the maximum RMS voltage did not exceed 1.2 kV.

**Fig 2:**
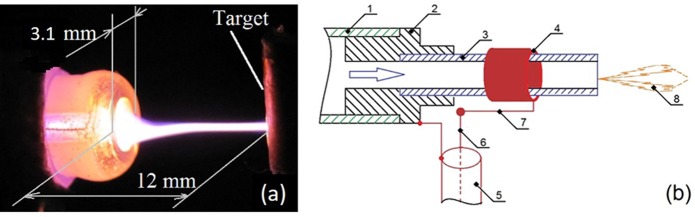
(a) External view of RF generator, plasma gun and gas power supply system. 1 –cylinder with working gas; 2 –reducer; 3 –gas channel; 4 –power cord; 5 –pulse generator; 6 –cable of plasma gun; 7 –plasma gun installed in dielectric optical bench; 8 –micrometric screw. (b) Plasma gun. 1 –flexible pipe (Tygon); 2 –stainless steel connector; 3 –dielectric tube (Pyrex or quartz); 4—ring electrode; 5 –coaxial cable; 6 –internal wire; 7 –connector; 8 –plasma plume.

The laboratory setup is shown in Fig 2A, and a sketch of the plasma gun is shown in Fig 2B. The CAPJ was generated inside a dielectric tube (Pyrex or quartz) by DBD RF discharge in Penning gas mixture (98% Ne+2%Ar) allowing decrease of the breakdown voltage threshold by several times as compared with other type of gases. RF pulses were applied to the ring-type electrode whose size and location with respect to the end of the dielectric capillary tube determine the efficiency of the plasma formation and its potential. Thus, only unshielded ring electrode is a source of electromagnetic radiation. In order to protect this electrode from Ohmic contact with the treated body, thin Capton film layers were used providing electrical insulation up to 10 kV against breakdown. Our resonant electronic scheme almost immediately (within one period of RF voltage, i.e. ≤1 μs) terminated the generation of high-voltage pulses in the case of shorting between the ring electrode and the treated body. Also, let us note that the replacement procedure of the plasma guns requires just several seconds. Several types of plasma gun (Fig 2), differing in length (10 – 30mm) and outer diameter of the dielectric tube (1.2 – 6mm), were designed and tested. As a result, we obtained generation of plasma plumes with different diameters (0.5–5 mm) and lengths (5–40 mm). Also, a sheet-like shape as well as multi-plasma plumes were generated using a rectangular tube and a multi-capillary tube. For the research described herein, a plasma gun with a total diameter (tube and cable together) of 3 mm was used at the end of a 150 cm-length coaxial cable.

Finally, the power delivered to the CAPJ was controlled using our electronic scheme. The latter allows to perform treatment of different targets with known value of the energy delivered to the plasma plume and to adjust the power delivered to the plume in the process of the experiments whenever it is necessary.

#### Cell lines

Murine squamous cell carcinoma (SCC-7), Colon cancer (DLD-1) and murine melanoma (B-16) cells were purchased from ATCC. FaDU hypopharyngeal cells were a king gift from Dr. Richard Wong of Memorial Sloan Kettering. The murine pancreatic adenocarcinoma cell line was created in our lab as previously described [36]. Cells were cultured in a medium containing 10% FBS and penicillin streptomycin.

#### In-Vitro CAPJ experiments

Cells were detached from culture dishes using trypsin, washed, and adjusted for 100,000 cells in 100μl PBS in a 1.5 Eppendorf. CAPJ was attached to a small animal stereotaxic apparatus (Kopf, model 963, CA, USA), and the tip adjusted to a height of 5mm above fluid level. CAPJ was then administered for designated time. Following CAPJ treatment, cells were plated in pentaplicates in 96 well plate. 24 hours after CAPJ treatment, 2,3-bis-(2-methoxy-4-nitro-5-sulfophenyl)-2H-tetrazolium-5-carboxanilide (XTT) assay was performed according to manufacturer’s instructions to access cellular proliferation (Biological Industries, Israel).

#### In-Vivo experiments

All animal experiments were approved by the institutional animal care and use committee at the Technion, authorization # IL-132-12-2012. C57/bl mice were purchased from Harlan. At the age of 6 weeks, mice fur was removed using small animal clipper, and 1 million melanoma cells were injected intra-dermally using a 2.5 μl Hamilton syringe (Hamilton, NV, USA). Mice were followed up routinely, and tumor size measured weekly by a caliper. Tumor volume was calculated as (L x W x H). In vivo imaging system (IVIS) measurements were done using the IVIS 200 imaging system (Perkin Elmer, MA, USA), with the following specifications: excitation: 445–490 nm, emission: 515–575 nm, background signal reduction: 410–440 nm. For CAPJ treatment, mice were under anesthesia using isoflurane. CAPJ was placed 1–2 mm above the skin, and treatment was done for designated time while continuously moving the probe to cover the entire tumor + a margin of 0.5 cm. For temperature measurements, a thermal visor Flir AX5 (CA, USA) was placed 20 cm above treated mouse. A Flir Tools application (CA, USA) was used to analyze data. CAPJ generation parameters for all the experiments, in-vitro and in-vivo, were: gas flow 3 l/min, RMS voltage of 1150 V, pulse duration of 800 μs repetition rate of 487 Hz.

## Results

### Plasma plume characterization

**Fig 3:**
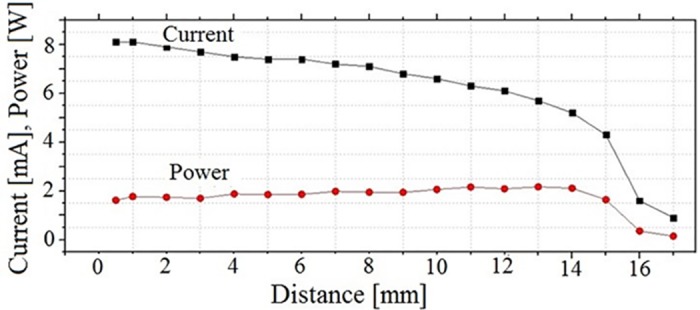
Dependence of the RF current amplitude and the power delivered to the plasma plume on the distance from the open end of the plasma gun having inner diameter of 1 mm. Gas flow rate Q = 0.5 l/min, RMS voltage of 750V, repetition rate of 222Hz, pulse duration of 450 μsec. In order to measure the temperature of the plasma plume and the target, a Ga-As thermometer (sensitivity of 0.1°C) and a thermal visor Flir AX5 [temperature range (20–42)°C] were used.

For characterization of the plasma plume, a copper target was placed at different distances from the open end of the plasma gun that was used. No significant difference in the plasma parameters was found when the target was either grounded or connected to the ground by a several kΩ resistor. A typical dependence of the RF current amplitude and the power delivered to the plasma relatively to the distance from the open end of the plasma gun is shown in Fig 3. One can see that the increase in the distance from 1 mm to 15 mm results in an almost two times decrease in the amplitude of the RF current and only an insignificant change in the delivered power. These data can be explained by the increase in the resistivity of the plasma plume versus its length.

**Fig 4:**
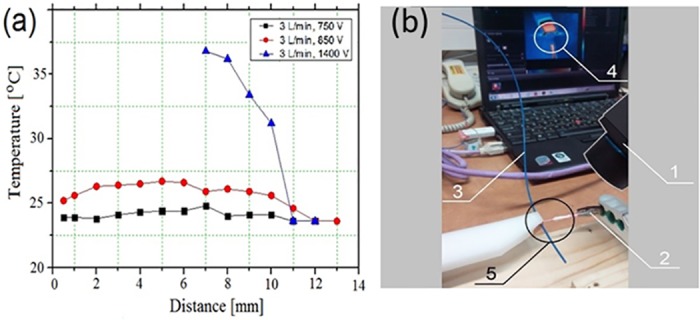
(a) Dependence of the temperature of the target on the distance between the open end of the plasma gun and the target. (b) Thermography of the CAPJ. 1 –thermal visor (Flir AX5), 2 –plasma gun; 3 –Ga-As thermometer’s cable; 4 –view of thermal visor output; 5 –plasma location (white circle on the thermal visor output corresponds to the black circle). Plasma gun with inner diameter of 3 mm. Gas flow rate of 3 L/min, RMS voltage 750/850/1400 V, repetition rate of 222 Hz, pulse duration of 450 μsec.

In general, it was found that the temperature of the target depends on the gas flow rate, the plasma plume diameter, the applied RF voltage and the duty cycle. Namely, an increase in the gas flow rate or plume diameter or a decrease in the duty cycle or voltage amplitude lead to a decrease in the temperature of the target. Typical dependencies of the target temperature on the distance of the target from the open end of the plasma gun are shown in Fig 4A for three values of the amplitude of the RF voltage. One can see that only at an RF voltage amplitude of 1400 V, the temperature of the target increases up to 36°C and in the case of smaller voltage amplitudes the temperature of the target is almost independent of the distance, remaining ≤ 27°C. In measurements made with the thermal visor (see Fig 4B) the plasma temperature appeared to be close to the ambient one. This is because no extra IR emission was measured in the gap between the plasma gun and the target.

**Fig 5:**
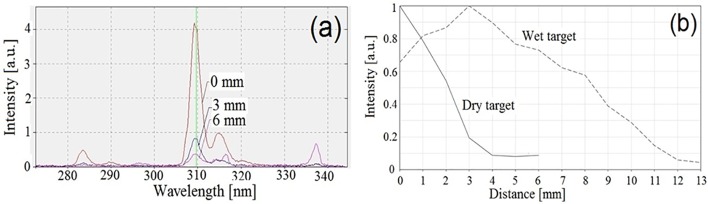
(a) Typical spectra of OH^—^band obtained at different distances from the open end of the plasma gun. (b) Relative intensity of OH^-^ (λ = 309.65 nm) versus the distance from the open end of the plasma gun. Plasma gun with inner diameter of 4.1mm, RMS voltage of 1150 V, repetition rate of 476 Hz, pulse duration of 800 μs; gas flow rate of 3 l/min; spectrometer exposure time of 100 ms, Al solid target.

A spectroscopic study of the plasma plume was carried out using an OceanOptics USB4000 spectrometer. Application of optical fiber, lenses and collimators allowed us to obtain spectra of the plasma plume light emission at different locations with respect to the open end of the plasma gun. In these experiments the target placed at different distances from the open end of the plasma gun, was either dry or wet [37,38]. In general, different spectral lines of excited hydrogen, oxygen, nitrogen were detected [39]. The main purpose of these experiments was observation of free radicals which could be important for treatment of cancer. Indeed, intense emission spectral line of excited OH^-^ radicals was obtained at different distances from the open end of the plasma gun. Typical example of this spectral band intensity and its dependence on the distance from the open end of the plasma gun are shown in Fig 5. One can see that in the case of dry target the intensity of the OH^-^ spectral line decreases rapidly and oppositely to the case of wet target where one obtains a rather greater uniformity of the OH^-^ spectral line intensity along almost 8 mm.

### CAPJ biological effect

In order to evaluate the effect of our novel CAPJ device on cancer cell proliferation in-vitro, we first applied plasma to different cancerous cell lines. 100,000 cells of each designated cell line were suspended in 100 μl of PBS, and CAPJ probe was placed 5 mm above the liquid surface.

**Fig 6:**
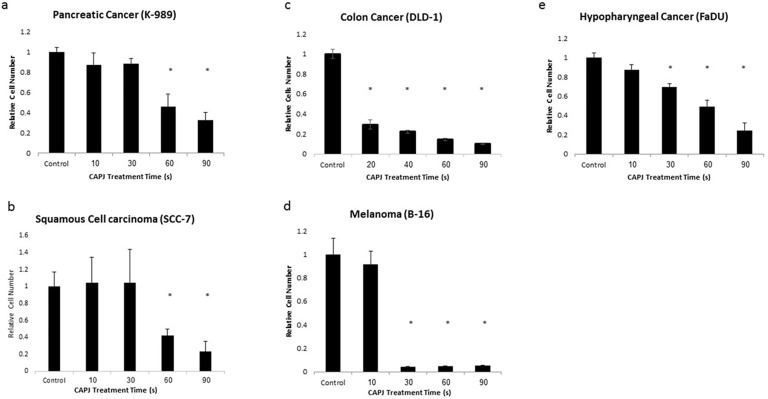
(a-e) Cells were treated with CAPJ for designated times, and plated in pentaplicates. 24 hours later proliferation was assessed by XTT. (p<0.05; p values were calculated by Student's t-test).

CAPJ was applied for different time durations, and as control, only gas flow was used while the CAPJ generation was turned off. Following treatment, cells were seeded in 96 well plates, and proliferation was evaluated 24 hours post treatment by XTT assay. Fig 6 shows that proliferation of all tested cell lines was significantly repressed by CAP treatment of 60 s and longer. While Pancreatic adenocarcinoma, skin squamous cell carcinoma and colon cancer were least sensitive to CAPJ treatment of 30 s, melanoma cells displayed extreme sensitivity to the treatment, with a mere 4% proliferation relative to control (p<0.001). This indicated, that like previously described CAPJ sources, our CAPJ device severely affects cancer cell proliferation in general, and melanoma cell proliferation in particular.

**Fig 7:**
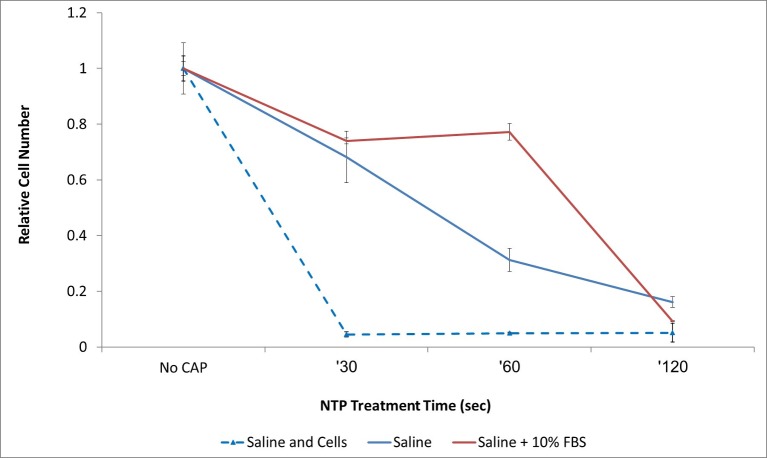
Effect of activated medium on cancer cell proliferation. Direct treatment of cells had better efficacy in proliferation reduction than activated medium (p<0.001 for all conditions). Protein addition to activated medium had a protective effect on the cells for 60 seconds of treatment (23% proliferation reduction vs. 69% for saline only (p<0.001).

Reports in the literature describe that CAPJ treatment to the culture medium alone, or directly to the cells cause cell death [11,40]. In order to verify that our novel CAPJ device can induce a similar effect, we applied CAPJ to two kinds of medium, with and without addition of protein (fetal bovine serum, FBS), and as controls, cells were treated in saline as described above. CAPJ was used for irradiation of medium for a designated amount of time, and after this, medium was transferred to cultures of melanoma cells for 4 hours. Medium was then aspirated, and normal culture medium put instead. 24 hours later, proliferation was assessed by XTT. Fig 7 demonstrates that indeed CAPJ-treated media could hamper cell proliferation, but is significantly less effective than direct treatment to cells of 30, 60 and 120 s (p<0.001 for three groups). Protein addition to treated media had a protective effect at 60 s, but at 120 s of treatment, saline with or without serum caused almost similar proliferation reduction (91% and 84% proliferation reduction respectively, p = 0.003).

**Fig 8:**
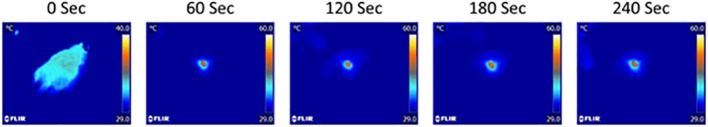
Wild type mouse was treated by CAPJ for 4 minutes. Each minute skin temperature was recorded by thermal visor. Experimental conditions: Gas flow 3 l/min, RMS voltage of 1150 V, pulse duration of 800 μs repetition rate of 487 Hz.

Before continuing to in-vivo experiments with melanoma tumors, we aimed to validate that CAPJ treatment does not cause thermal damage to treated tissue. To this end, we used a thermal visor to measure skin temperature of CAPJ treated mouse. The mouse was treated for 4 minutes, and skin temperature was captured in the beginning of the experiment, and each minute later. Fig 8 demonstrates that maximal skin temperature was 50°C at 180 s of treatment, and that the temperature did not rise over the time course of the experiment. We concluded that CAPJ treatment could be safely administered to skin surfaces for short durations, without fear of causing thermal damage to the tissue.

**Fig 9:**
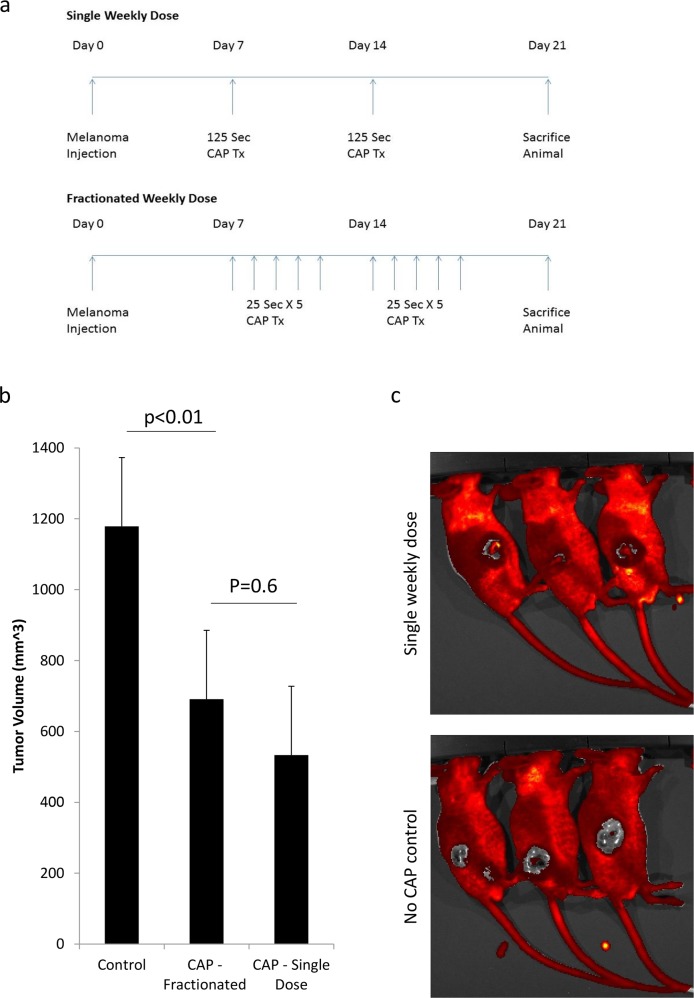
(a) Experiment scheme. Two experimental arms: single weekly dose, and fractionated therapy. Both arms had the same overall CAPJ treatment duration. Experimental conditions: Gas flow 3 l/min, RMS voltage of 1150 V, pulse duration of 800 μs repetition rate of 487 Hz. (b) Tumor volume measurement for the three groups at the end of the experiment. (p values were calculated by student’s t-test). (c) IVIS image of control mice, and single arm treatment mice. Melanoma tumor is seen in grey.

Next we aimed to evaluate the efficacy of our CAPJ source on melanoma tumors in-vivo. Here we compared two CAPJ treatment regimens of two weeks, with identical overall CAPJ dose. In the single weekly dose, 125 s of CAPJ were applied once weekly, and in the fractionated arm, 25 s of treatment were applied at five consecutive days a week (N = 3 per group) (Fig 9A). Treatment was initiated one week following a flank intradermal injection of 1 million B-16 murine melanoma cells, when melanoma tumor larger than 1 mm in diameter was evident on the skin. As controls, mice were injected with melanoma but not treated with CAPJ. At the end of the study animals were scanned using IVIS with a green fluorescence protein (GFP) filter. This filter was chosen due to its ability to distinguish between the light-absorbing melanotic tumors and their GFP emitting surrounding [41]. Finally, mice were sacrificed, and the tumors were measured and processed for pathology.

Measurement of the tumors demonstrated that CAPJ treatment caused significant reduction in tumor volume in both treatment arms (41.4% for single therapy arm and 54.7% tumor volume reduction in the fractionated arm compared to control, p<0.001). However, the difference between the two groups was not significant (p = 0.6) (Fig 9B). IVIS measurement strengthened these results, showing that melanoma penetration into the epidermal layer was reduced by 48.2% in the single-dose group relatively to controls (120,868 pixels as compared to 62,608 pixels) (Fig 9C).

**Fig 10:**
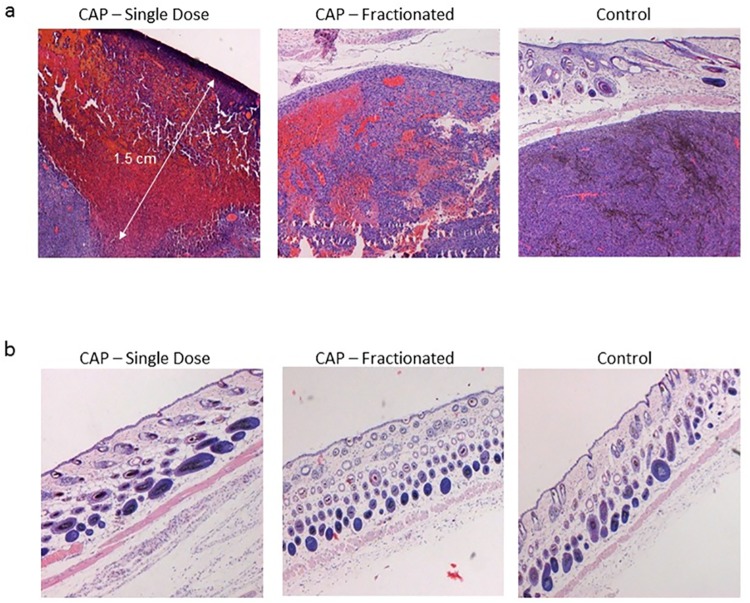
H&E images (a) of a representative tumor in the single, fractionated and control treatment arms. (b) of mice in the skin control group, treated by the three regiments.

Fig 10A depicts H&E staining of the melanoma tumors. In the single dose arm, a confined area of necrotic tumor was seen, with a maximal depth of 15 mm from skin surface. In the fractionated treatment arm, necrotic areas were evident, but were more sporadic. No necrotic areas were seen in the untreated control group. In order to examine CAPJ effect on normal skin, three groups of mice (N = 3 per group) were assigned to receive single weekly dose or fractionated CAPJ therapy as above. Controls were not treated by CAPJ. Pathological examination of skin specimens from mice in the three groups demonstrated no crude abnormality of all dermal layers (Fig 10B).

## Discussion

The incidence of cutaneous melanoma, a malignancy notoriously known for being resistant to therapy, is increasing at 2–5% per year, and disproportionately targets young individuals. The cornerstone of treatment of melanoma remains surgical excision, although alternative therapeutic options continue to improve with time. Because up to 29% of cases initially diagnosed as melanoma in-situ (confined to the epidermis) are upstaged to invasive malignant melanoma after pathology review, local excision with margin of at least 0.5–0.6 cm is recommended. In some cases, wide margin excision cannot be achieved due to proximity to vital anatomical structures such as nerves or blood vessels, or when severe cosmetic issues evolve, especially in the head and neck region. In these cases second-line treatments as topical agents, intralesional alpha-interferon, radiation, and laser therapy are being explored as adjuncts to surgery, although their efficacy is not yet undoubtedly proven [28]. For these patients, for whom wide margin excision could not be achieved, CAPJ therapy might prove to be an appealing alternative.

Few studies investigated the use of CAPJ against melanoma, all of them were performed in-vitro. Despite being preliminary, all have demonstrated an anti-proliferative effect of CAPJ on melanoma cells. Lee, Kim and their colleagues, have demonstrated that CAPJ caused apoptosis in melanoma cells, which is accompanied by loss of integrins and focal adhesion kinase from the cells membrane [31,32]. Ishaq *et al*. showed that CAPJ causes melanoma cells to over-express TP73, leading to growth arrest and apoptosis [29]. Importantly, Ardnt *et al*. showed that 2 minutes of CAPJ treatment caused irreversible cell cycle arrest, and induction of phosphor-H2AX, indicative of DNA damage [19]. In line with these previous data, our in-vitro results demonstrate that melanoma cells are extremely sensitive to CAPJ treatment, and as little as 30 seconds can reduce cell proliferation in more than 90% (Fig 6). Kurake *et al*. have proposed that H_2_O_2_ and NO_2_^-^ generated in cell medium following CAPJ treatment are sufficient to cause cell cycle arrest [40]. Our experiments indicate that indeed, CAPJ-treated-medium is toxic to melanoma cells, but significantly less than the direct effect of CAPJ. Moreover, we show that addition of proteins to CAPJ activated media reduces its toxicity (Fig 7), probably due to increased decay of radicals in protein rich environment relatively to saline alone. Few studies investigated the use of CAP against melanoma, all of them were performed in-vitro. Despite being preliminary, all have demonstrated an anti-proliferative effect of CAP on melanoma cells. Lee, Kim and their colleagues, have demonstrated that CAP caused apoptosis in melanoma cells, which is accompanied by loss of integrins and focal adhesion kinase from the cells membrane [31,32]. Ishaq *et al*. showed that CAP causes melanoma cells to over-express TP73, leading to growth arrest and apoptosis [29]. Importantly, Ardnt and Kalghatgi showed that CAP treatment caused irreversible cell cycle arrest, and induction of phosphor-H2AX, indicative of DNA damage [19,27]. In line with these previous data, our in-vitro results demonstrate that melanoma cells are extremely sensitive to CAP treatment, and as little as 30 seconds can reduce cell proliferation in more than 90% (Fig 6). Kurake *et al*. have proposed that H_2_O_2_ and NO_2_^-^ generated in cell medium following CAP treatment are sufficient to cause cell cycle arrest [40]. Our experiments indicate that indeed, CAP-treated-medium is toxic to melanoma cells, but significantly less than the direct effect of CAP. Moreover, we show that addition of proteins to CAP activated media reduces its toxicity (Fig 7), probably due to increased decay of radicals in protein rich environment relatively to saline alone.

Preliminary data suggests that CAPJ has selective-toxicity towards cancer cells rather than non-neoplastic cells, but the mechanism that leads to CAPJ selectivity is poorly understood. Some researchers attribute the selective effect of CAPJ to lower reserves of anti-oxidants in cancer cells, which cannot oppose reactive oxygen and nitrogen species (ROS and RNS) [42]. In turn, ROS and RNS lead to DNA damage that hampers cellular proliferation, much like the effect of conventional radiation therapy. Although melanoma was traditionally considered as radio-insensitive, it has been reputedly demonstrated that melanoma is sensitive to high-dose per fraction radiation [43,44]. Inspired by these data, we have planned our in-vivo experiments to demonstrate whether melanoma is more susceptible to high-dose per fraction CAPJ treatment, like radiotherapy. Although not statistically significant, our experiments demonstrate a clear trend of higher efficacy of CAPJ treatment when provided in high-dose per fraction (Fig 9). The ideal method for destruction of cancer cells is the induction of apoptosis, rather than necrosis which inflicts sever pain and immediate inflammatory response. Supporting this is our observation that response to CAPJ treatment takes 24–72 hours to develop (no immediate burn, blister, bleeding or edema are seen in the treatment site), indicating apoptotic process. Only later does tissue damage begin to be evident. Pathology specimens presented in Fig 10 (that were taken 1 week following treatment cessation) further strengthen our observation, showing a confined area of tumor necrosis in the high-dose per fraction treatment arm, relatively to sporadic necrosis in the low-dose per fraction arm. Taken together, these results might indicate that CAPJ and radiotherapy share some similarities in their mechanism of action, possibly by causing ROS-dependent DNA damage. The role of ROS and RONs and their contribution to plasma selectivity, however, was not addressed in this study. Future research should examine whether boosting the amount of radical scavengers (like N-Acetyl-Cysteine) protects cancer cells from CAPJ-induced apoptosis, and whether low anti-oxidant reserve (such as glutathione) explain melanoma cell susceptibility to CAPJ.

The potential use of CAPJ technologies in medicine has been addressed in the literature for the last decade, but disappointingly, little progress was made to bring this technology to the bedside. While crucial safety issues that have yet been addressed might explain the slow adoption of this technology, a main problem remains to be the design of CAPJ devices to be suitable for medical use. Bearing in mind pre-requests for successful clinical adoption, we designed our CAPJ device to be narrow, long and flexible, enabling the operator to direct the CAPJ plume directly to the treatment location with maximal precision. The electric and gas cords connecting the generator with the CAPJ probe are long, so that the power generator could be placed afar from the patient, leaving the treatment sight visible and approachable. Importantly, the unique electrical properties of our CAPJ device does not endanger the patient or the operator with high current electricity.

In conclusion, our results demonstrate the efficacy of CAPJ treatment against melanoma in- vitro and in-vivo. Our new approach to create CAPJ at the tip of an elongated flexible capillary using low electrical currents, might pave the way for new opportunities to use CAPJ in the field of oncology.

## References

[1] Graves DB. Low temperature plasma biomedicine: A tutorial reviewa). Phys Plasmas. AIP Publishing; 2014;21: 80901.

[2] Fridman G, Shereshevsky A, Jost MM, Brooks AD, Fridman A, Gutsol A, et al Floating electrode dielectric barrier discharge plasma in air promoting apoptotic behavior in Melanoma skin cancer cell lines. Plasma Chem Plasma Process. 2007;27: 163–176.

[3] Shashurin A, Shneider MN, Keidar M. Measurements of streamer head potential and conductivity of streamer column in cold nonequilibrium atmospheric plasmas. Plasma Sources Sci Technol. 2012;21: 49601. 10.1088/0963-0252/21/3/034006 PMC4309986 25642104

[4] Walsh JL, Olszewski P, Bradley JW. The manipulation of atmospheric pressure dielectric barrier plasma jets. Plasma Sources Sci Technol. 2012;21: 34007.

[5] Weltmann K-D, Kindel E, Brandenburg R, Meyer C, Bussiahn R, Wilke C, et al Atmospheric Pressure Plasma Jet for Medical Therapy: Plasma Parameters and Risk Estimation. Contrib to Plasma Phys. 2009;49: 631–640.

[6] Choi EH. Diagnostics of Nonthermal Atmospheric Pressure Plasma for Plasma Biosciences and their Biological Cell Interactions throughout Ultraviolet Photolysis. Bull Am Phys Soc. American Physical Society; 2015;Volume 60,. Available: http://meetings.aps.org.ezlibrary.technion.ac.il/link/BAPS.2015.GEC.WF4.2

[7] Wang M, Holmes B, Cheng X, Zhu W, Keidar M, Zhang LG. Cold atmospheric plasma for selectively ablating metastatic breast cancer cells. PLoS One. 2013;8: e73741 10.1371/journal.pone.0073741 24040051 PMC3770688

[8] Keidar M, Shashurin A, Volotskova O, Ann Stepp M, Srinivasan P, Sandler A, et al Cold atmospheric plasma in cancer therapy. Phys Plasmas. 2013;20.

[9] Judée F, Fongia C, Ducommun B, Yousfi M, Lobjois V, Merbahi N. Short and long time effects of low temperature Plasma Activated Media on 3D multicellular tumor spheroids. Sci Rep. Nature Publishing Group; 2016;6: 21421 10.1038/srep21421 26898904 PMC4761900

[10] Yousfi M, Eichwald O, Merbahi N, Jomaa N, Stoffels E KIESRE J van den BLJM van der LEP and SM M L, et al Analysis of ionization wave dynamics in low-temperature plasma jets from fluid modeling supported by experimental investigations. Plasma Sources Sci Technol. IOP Publishing; 2012;21: 45003.

[11] Tanaka H, Mizuno M, Ishikawa K, Takeda K, Hashizume H, Nakamura K, et al Responses of cells in plasma-activated medium. Bull Am Phys Soc. APS; 2015;60.

[12] Plewa J-M, Yousfi M, Frongia C, Eichwald O, Ducommun B, Merbahi N, et al Low-temperature plasma-induced antiproliferative effects on multi-cellular tumor spheroids. New J Phys. IOP Publishing; 2014;16: 43027.

[13] Florian J, Merbahi N, Yousfi M. Genotoxic and Cytotoxic Effects of Plasma-Activated Media on Multicellular Tumor Spheroids. Plasma Med. Begel House Inc.; 2016;6: 47–57.

[14] Graves DB. Low temperature plasma biomedicine: A tutorial reviewa) Low temperature plasma biomedicine: A tutorial review a). 2014;80901: 0–12.

[15] Kieft IE, Broers JL V, Caubet-Hilloutou V, Slaaf DW, Ramaekers FCS, Stoffels E. Electric discharge plasmas influence attachment of cultured CHO K1 cells. Bioelectromagnetics. 2004;25: 362–8. 10.1002/bem.20005 15197760

[16] Volotskova O, Hawley TS, Stepp M a, Keidar M. Targeting the cancer cell cycle by cold atmospheric plasma. Sci Rep. 2012;2: 636 10.1038/srep00636 22957140 PMC3434394

[17] Weiss M, Gümbel D, Hanschmann E-M, Mandelkow R, Gelbrich N, Zimmermann U, et al Cold Atmospheric Plasma Treatment Induces Anti-Proliferative Effects in Prostate Cancer Cells by Redox and Apoptotic Signaling Pathways. PLoS One. 2015; 10.1371/journal.pone.0130350 PMC4488447 26132846

[18] Brullé L, Vandamme M, Riès D, Martel E, Robert E, Lerondel S, et al Effects of a non thermal plasma treatment alone or in combination with gemcitabine in a MIA PaCa2-luc orthotopic pancreatic carcinoma model. PLoS One. 2012;7: e52653 10.1371/journal.pone.0052653 23300736 PMC3530450

[19] Arndt S, Wacker E, Li YF, Shimizu T, Thomas HM, Morfill GE, et al Cold atmospheric plasma, a new strategy to induce senescence in melanoma cells. Exp Dermatol. 2013; 10.1111/exd.12127 23528215

[20] Köritzer J, Boxhammer V, Schäfer A, Shimizu T, Klämpfl TG, Li YF, et al Restoration of Sensitivity in Chemo—Resistant Glioma Cells by Cold Atmospheric Plasma. PLoS One. 2013;8. 10.1371/journal.pone.0064498 PMC3660344 23704990

[21] Walk RM, Snyder JA, Srinivasan P, Kirsch J, Diaz SO, Blanco FC, et al Cold atmospheric plasma for the ablative treatment of neuroblastoma. J Pediatr Surg. 2013; 10.1016/j.jpedsurg.2012.10.020 23331795

[22] Kim JY, Ballato J, Foy P, Hawkins T, Wei Y, Li J, et al Apoptosis of lung carcinoma cells induced by a flexible optical fiber-based cold microplasma. Biosens Bioelectron. Elsevier B.V.; 2011;28: 333–338. 10.1016/j.bios.2011.07.039 21820891

[23] Schlegel J, Köritzer J, Boxhammer V. Plasma in cancer treatment. Clin Plasma Med. Elsevier; 2013;1: 2–7.

[24] Kim C-H, Bahn JH, Lee S-H, Kim G-Y, Jun S-I, Lee K, et al Induction of cell growth arrest by atmospheric non-thermal plasma in colorectal cancer cells. J Biotechnol. Elsevier B.V.; 2010;150: 530–538. 10.1016/j.jbiotec.2010.10.003 20959125

[25] Keidar M, Walk R, Shashurin a, Srinivasan P, Sandler a, Dasgupta S, et al Cold plasma selectivity and the possibility of a paradigm shift in cancer therapy. Br J Cancer. Nature Publishing Group; 2011;105: 1295–301. 10.1038/bjc.2011.386 21979421 PMC3241555

[26] Weltmann K-D, von Woedtke T. Basic requirements for plasma sources in medicine. Eur Phys J Appl Phys. 2011;55: 13807.

[27] Kalghatgi S, Kelly CM, Cerchar E, Torabi B, Alekseev O, Fridman A, et al Effects of Non-Thermal Plasma on Mammalian Cells. Koutsopoulos S, editor. PLoS One. Public Library of Science; 2011;6: e16270 10.1371/journal.pone.0016270 21283714 PMC3025030

[28] Higgins HW, Lee KC, Galan A, Leffell DJ. Melanoma in situ: Part II. Histopathology, treatment, and clinical management. J Am Acad Dermatol. Elsevier Inc; 2015;73: 193–203. 10.1016/j.jaad.2015.03.057 26183968

[29] Ishaq M, Bazaka K, Ostrikov K. Intracellular effects of atmospheric-pressure plasmas on melanoma cancer cells. Phys Plasmas. 2015;22: 122003.

[30] Kim JY, Wei Y, Li J, Kim SO. 15-Μm-Sized Single-Cellular-Level and Cell-Manipulatable Microplasma Jet in Cancer Therapies. Biosens Bioelectron. Elsevier B.V.; 2010;26: 555–559. 10.1016/j.bios.2010.07.043 20685106

[31] Kim G-C, Lee HJ, Shon C-H. The Effects of a Micro plasma on Melanoma (G361) Cancer Cells. J Korean Phys Soc. 2009;54: 628.

[32] Lee HJ, Shon CH, Kim YS, Kim S, Kim GC, Kong MG. Degradation of adhesion molecules of G361 melanoma cells by a non-thermal atmospheric pressure microplasma. New J Phys. 2009;11.

[33] Zucker SN, Zirnheld J, Bagati A, DiSanto TM, Des Soye B, Wawrzyniak JA, et al Preferential induction of apoptotic cell death in melanoma cells as compared with normal keratinocytes using a non-thermal plasma torch. Cancer Biol Ther. 2012;13: 1299–1306. 10.4161/cbt.21787 22895073 PMC3493438

[34] Robert E, Vandamme M, Brullé L, Lerondel S, Le Pape a., Sarron V, et al Perspectives of endoscopic plasma applications. Clin Plasma Med. Elsevier; 2013;1: 8–16.

[35] Raizer YP. Gas discharge physics Springer Verlag Berlin; 1987.

[36] Weizman N, Krelin Y, Shabtay-Orbach a, Amit M, Binenbaum Y, Wong RJ, et al Macrophages mediate gemcitabine resistance of pancreatic adenocarcinoma by upregulating cytidine deaminase. Oncogene. Nature Publishing Group; 2013; 1–8. 10.1038/onc.2013.357 23995783

[37] Miichi T, Ihara S, Satoh S, Yamabe C. Spectroscopic measurements of discharges inside bubbles in water. Vacuum. 2000;59: 236–243.

[38] Kornev J, Yavorovsky N, Preis S, Khaskelberg M, Isaev U, Chen B-N. Generation of Active Oxidant Species by Pulsed Dielectric Barrier Discharge in Water-Air Mixtures. Ozone Sci Eng. 2006;28: 207–215.

[39] NIST: Atomic Spectra Database Lines Form [Internet]. [cited 23 May 2016]. Available: http://physics.nist.gov/PhysRefData/ASD/lines_form.html

[40] Kurake Naoyuki, Tanaka Hiromasa, Ishikawa Kenji, Nakamura Kae, Kajiyama Hiroaki, Kikkawa Fumiaki, et al. Antitumor effect of synergistic contribution of nitrite and hydrogen peroxide in the plasma activated medium. APS Gaseous Electron Conf 2015. 2015; Available: http://adsabs.harvard.edu/abs/2015APS..GECFT1007K

[41] Andersson H, Kim Y-S, O’Neill B, Shi Z-Z, Serda R. HSP70 Promoter-Driven Activation of Gene Expression for Immunotherapy Using Gold Nanorods and Near Infrared Light. Vaccines. 2014;2: 216–227. 10.3390/vaccines2020216 25328682 PMC4199457

[42] Graves DB. The emerging role of reactive oxygen and nitrogen species in redox biology and some implications for plasma applications to medicine and biology. J Phys D Appl Phys. 2012;45: 263001.

[43] Fuji H, Yoshikawa S, Kasami M, Murayama S, Onitsuka T, Kashiwagi H, et al High-dose proton beam therapy for sinonasal mucosal malignant melanoma. Radiat Oncol. 2014;9: 162 10.1186/1748-717X-9-162 25056641 PMC4118609

[44] Khan N, Khan MK, Almasan A, Singh AD, MacKlis R. The evolving role of radiation therapy in the management of malignant melanoma. Int J Radiat Oncol Biol Phys. 2011;80: 645–654. 10.1016/j.ijrobp.2010.12.071 21489712 PMC3119033

